# C_4_ grasses employ distinct strategies to acclimate rubisco activase to heat stress

**DOI:** 10.1042/BSR20240353

**Published:** 2024-10-23

**Authors:** Sarah C. Stainbrook, Lindsey N. Aubuchon, Amanda Chen, Emily Johnson, Audrey Si, Laila Walton, Angela J. Ahrendt, Daniela Strenkert, Joseph M. Jez

**Affiliations:** 1 Department of Biology, Washington University in St Louis, St Louis, MO, USA; 2 Plant Research Laboratory, Michigan State University, East Lansing, MI, USA; 3 Illinois Mathematics and Science Academy, Aurora, IL, USA

**Keywords:** abiotic stress, acclimation, C4 photosynthesis, heat stress, rubisco activase

## Abstract

Rising temperatures due to the current climate crisis will soon have devastating impacts on crop performance and resilience. In particular, CO_2_ assimilation is dramatically limited at high temperatures. CO_2_ assimilation is accomplished by rubisco, which is inhibited by the binding of inhibitory sugar phosphates to its active site. Plants therefore utilize the essential chaperone rubisco activase (RCA) to remove these inhibitors and enable continued CO_2_ fixation. However, RCA does not function at moderately high temperatures (42°C), resulting in impaired rubisco activity and reduced CO_2_ assimilation. We set out to understand temperature-dependent RCA regulation in four different C_4_ plants, with a focus on the crop plants maize (two cultivars) and sorghum, as well as the model grass *Setaria viridis* (setaria) using gas exchange measurements, which confirm that CO_2_ assimilation is limited by carboxylation in these organisms at high temperatures (42°C). All three species express distinct complements of RCA isoforms and each species alters the isoform and proteoform abundances in response to heat; however, the changes are species-specific. We also examine whether the heat-mediated inactivation of RCA is due to biochemical regulation rather than simple thermal denaturation. We reveal that biochemical regulation affects RCA function differently in different C_4_ species, and differences are apparent even between different cultivars of the same species. Our results suggest that each grass evolved different strategies to maintain RCA function during stress and we conclude that a successful engineering approach aimed at improving carbon capture in C_4_ grasses will need to accommodate these individual regulatory mechanisms.

## Introduction

Ribulose-1,5-bisphosphate carboxylase/oxygenase (rubisco) is the most abundant protein on earth and is responsible for almost all carbon fixation on Earth. Rubisco catalyzes the addition of CO_2_ to the C5 sugar ribulose-1,5-bisphosphate (RuBP), producing two molecules of glycerate-3-phosphate, which are subsequently further assimilated in the Calvin–Benson–Bassham (CBB) cycle. Rubisco is an ancient but inefficient enzyme, assimilating only about 3–10 CO_2_ molecules per second, and it lacks specificity between carbon (CO_2_) and oxygen (O_2_). When rubisco mistakenly fixes O_2_ rather than CO_2_, 2-phosphoglycolate is produced instead of glycerate-3-phosphate. The photorespiratory pathway is used to remediate this biochemical error, causing the release of one molecule of CO_2_ for each O_2_ fixed and further reducing of the efficiency of CO_2_ assimilation [1]. Rubisco is also inhibited by the binding of sugar phosphates to the active site in place of the RuBP substrate, which inhibits rubisco enzyme activity until the inhibitor is removed [2]. Rubisco activase (RCA) facilitates the removal of inhibitory substrates and thereby restores rubisco activity, rendering this protein essential for photosynthetic carbon fixation. However, RCA itself is not without issues, especially with regard to thermotolerance; its activity is inhibited by even moderate heat (i.e. 32.5°C in maize) [3–5] and this inhibition limits the overall heat tolerance of photosynthesis [6,7]. RCA’s inability to function properly at higher temperatures thus has grave implications for crop yield, especially given the rise in global average temperatures and the increased occurrence of heat waves.

### RCA structure and function

RCA is a highly conserved AAA-family ATPase which functions in a hexameric ring structure [8]. Most plant species express two RCA isoforms, α and β, which can arise either from alternative splicing of a single gene (e.g. spinach, Arabidopsis) or from two dedicated genes (e.g. maize, setaria, and sorghum). The β isoform is approximately 43 kDa in size and can undergo post-translational proteolytic cleavage to form a 41 kDa proteoform in maize [9,10]. In most species, the β isoform is expressed constitutively. The α isoform is slightly bigger, approximately 45 kDa, and is mostly identical with the β isoform with the addition of a C-terminal domain (CTD), which is the predominant site of biochemical regulation. The α and β monomers can differ in thermostability, with the α isoform frequently being reported as more thermostable [11]. The thermolability of RCA may be due to structural changes such as denaturation and aggregation [5,12]. However, RCA activity is also regulated by other factors inside the chloroplast.

### RCA regulation

The transcript stability and protein abundance of RCA are known to be regulated in response to heat stress [13–15]. The expression of the α isoform in particular is frequently upregulated in response to stress [11,15–17]. Because the α and β isoforms interact to create hetero-oligomers, changes in the relative amounts of α and β isoforms can alter the overall properties of the RCA pool within the cell [18,19]. However, the abundance of RCA alone is only sometimes adequate to explain the activation state of rubisco [9,20], since RCA is often present in excess during optimal steady-state conditions [21] suggesting additional factors can regulate the activity of RCA.

RCA activity is also affected by the biochemical environment of the chloroplast stroma. The CTD of the α isoform contains two cysteines capable of forming a redox-sensitive disulfide bond, coupling RCA activity to the redox state of the chloroplast, which is dynamically modulated by illumination [22]. This redox-mediated change likely occurs by changing the binding affinity between RCA and ATP [23,24]. The ATP/ADP ratio is a measure of the status of photosynthetic electron transport in the chloroplast, and the relative concentrations of ATP and ADP can also affect RCA function in some species [12,25,26]. In the α isoform, this sensitivity appears to be modulated by negatively charged residues in the CTD [23]. However, sensitivity to elevated ADP is also observed in tobacco which lacks the α isoform and therefore lacks the CTD [22,25].

Recent evidence indicates that the activity of RCA is related to its oligomeric state and that biochemical regulation on RCA may occur by means of modulating oligomerization [12,18,19,27–33]. In the cell, RCA exists in three pools: inactive dimers, active hexamers, and inactive, thermostable higher-order aggregates. To function, RCA must form hexamers, catalyze hydrolysis of ATP (which can be coupled to rubisco activation), then dissociate into dimers, exchange ADP for ATP, and reform hexamers on the time scale of about a minute [19]. Hexamer formation alone is not sufficient for activity; rather, the dynamic cycling of subunits between hexamer and dimer pools is required for activity [31]. RCA transitions between hexamer and dimer pools involve the formation and breaking of inter-subunit contacts, the affinity and stability of which are determined by subunit concentration and modulated by the concentration of ATP, ADP, and Mg^2+^ [12,27–29,32]. Additionally, α and β RCA isoforms can form hetero-oligomeric complexes, and the thermostability and activity of the entire functional complex is thereby influenced by the conditions affecting both isoforms [19,33].

The mechanisms governing heat-induced inactivation of RCA remain unknown. Simple thermal denaturation provides an attractive explanation, but *in vitro* measurements of RCA denaturation indicate a higher melting temperature for RCA than its observed loss of function *in vivo* [12]. We therefore examined other regulation that are known to affect RCA activity and asked whether the heat inactivation may be mediated by these biochemical changes.

### C_4_ RCA as an engineering target

RCA’s rapid heat inactivation makes it an attractive target for engineering interventions designed to improve plants’ resilience to heat [34]. However, rubisco’s propensity toward mis-fixation of O_2_ increases with rising temperatures, leading to a correspondingly increased cost of photorespiration [35]. Therefore, some have proposed that the thermolability of RCA in C_3_ plants is in fact a protective feature that disables photosynthesis under conditions where photorespiration becomes too high, similar to a fuse [19,36,37]. Tight-binding inhibitors can then protect rubisco from oxidative damage and protease degradation [38]. If this is true, producing a thermostable RCA in C_3_ plants would not improve CO_2_ fixation at high temperatures, but only allow higher photorespiratory flux.

However, C_4_ plants have evolved to minimize photorespiration. In C_4_ plants, rubisco is sequestered into bundle sheath cells away from the primary site of PSII-dependent O_2_ production but receives concentrated CO_2_ from mesophyll cells through a pumping mechanism. This adaptation limits photorespiration to less than a fifth of that of comparable C_3_ grasses [39]. *In vitro*, the turnover speed of fully active rubisco improves with increasing temperature, hinting that C_4_ plants could photosynthesize more efficiently at higher temperatures if RCA could maintain rubisco activity *in vivo* [7,40]. However, the thermolability of RCA limits CO_2_ assimilation in C_4_ plants, and inactivation of rubisco by the binding of inhibitory sugar phosphates remains the predominant reason for reduced CO_2_ assimilation at elevated temperatures. The RCA of C_4_ plants is therefore an attractive target for protein engineering with the goal of improving its thermostability and thereby to increase CO_2_ assimilation when plants experience high temperatures [34].

Efforts to discover or design thermostable RCA isoforms have been advocated widely and have already shown moderate success [41–47]. To date, these efforts have not explicitly considered possible regulatory effects of stromal components or post-translational modifications on either native or synthetic enzymes, making it likely that potential pitfalls and promising avenues for improvement have been missed. This is mostly due to an incomplete understanding of RCA regulation itself and in response to changes in its native environment: the dynamically changing chloroplast stroma. Toward this end, we examined whether CO_2_ assimilation is truly limited by RCA during heat in the C_4_ food crops *Zea mays* (maize), *Sorghum bicolor* (sorghum) and in the common C_4_ model plant *Setaria viridis* (setaria). These species each possess α and β isoforms produced by dedicated genes. Intraspecific variation was assessed among several cultivars of maize, and interspecific variation was examined by comparison with sorghum and setaria. RCA isoform expression was determined in each species, and we examined how stromal conditions that are known to regulate RCA function may alter its activity during heat treatment. Our results expose potential avenues for improvement of RCA with the long-term goal to bioengineer crop species that maintain photosynthetic performance despite rising global temperature.

## Materials and methods

### Plant material

*S. viridis* A10 was a gift from the lab of Dr. Ivan Baxter at the Donald Danforth Plant Science Center in St. Louis. Seeds were sown 1 cm deep in 18-well trays in Berger Bark Mix and thinned to one plant per cell. Plants were grown in the Washington University in St Louis greenhouse with 28°C/22°C temperatures and a 16-h photoperiod. Supplemental LED or halogen lighting maintained light intensity at or above 600 μE/m^2^s. *Z. mays* B73 was obtained from the greenhouse at Washington University in St Louis. All other maize accessions were obtained from the USDA GRIN catalog. *S. bicolor* BT × 623 was a gift from the lab of Dr. Malia Gehan at the Donald Danforth Plant Science Center. Sorghum and maize were planted 2 cm deep in a 4-inch pot in a 50% mixture of Berger Bark Mix and Turface and grown in the Washington University in St Louis greenhouse with 28°C/22°C temperatures and a 16-h photoperiod. Plants were grown to the two- or three-leaf stage, then transplanted into 6-inch pots in the same soil mix.

### Heat treatment

Heat response measurements were performed on plants with at least six fully expanded leaves. Greenhouse-grown plants were moved into a growth chamber of the appropriate temperature (42°C for most measurements) and 1000 μmol m^−2^ s^−1^ light intensity. Chambers were kept at 50% humidity and plants were maintained with the bottom of the pot in standing water to avoid drought stress. For 1-h measurements, the leaf was acclimated in the 2 cm^2^ leaf chamber fluorometer of the LI-6400XT instrument with the instrument controlling the leaf temperature. For longer measurements, the gas exchange chamber was attached to the leaf 20 min prior to the measurement to allow stomata to equilibrate. For 48-h measurements, plants were maintained at the same photoperiod as the greenhouse with 42°C day and 38°C night temperatures. All heat treatments were initiated, and all gas exchange measurements were taken at least 2 h from either end of the photoperiod.

### Gas exchange measurements

Gas exchange measurements were performed on the most recently fully expanded leaf as defined by ligule development. The leaf measured was from the main stem of each plant, and all measurements were taken prior to panicle emergence. CO_2_ concentration within the gas exchange chamber was controlled at 400 ppm. A light saturation curve (Supplementary Figure S1) was performed on each species and the saturating illumination was determined to be 1400 μmol m^−2^ s^−1^ for setaria and 2000 μmol m^−2^s^−1^ for maize and sorghum. All subsequent gas exchange measurements were performed at saturating illumination. Humidity was controlled to maintain stomatal conductance above 0.3 for all measurements. The same plants were measured at the 1, 6, and 48 h time points whenever feasible. In our greenhouse-grown setaria, a single leaf filled the entire 2 cm^2^ chamber when positioned carefully. Correction for area was therefore not needed.

A/Ci curve measurements were taken according to the standard method [48]. Briefly, the leaf was clamped in the chamber and allowed to equilibrate at the treatment temperature (leaf temperature maintained at 25°C or 42°C), flow rate of 500 μmol s^−1^, 400 μM CO_2_, and saturating illumination for at least 20 min or until a stable assimilation value was reached. For the 1-h measurements, the heat treatment was performed with the leaf clamped for the entire hour of acclimation. The CO_2_ concentration in the chamber was then decreased stepwise to 300, 250, 200, 150, 100, 50, and 0 μM. At each step, matching was performed, and the assimilation value was allowed to stabilize for a maximum of 180 s before assimilation was measured. Low CO_2_ concentrations can cause changes in rubisco activation state and stomatal conductance, so it is important that these low-CO_2_ measurements be performed as rapidly as possible to avoid significant effects of these changes on assimilation [48]. The CO_2_ concentration was returned to 400 μM and maintained until assimilation returned to its initial value to ensure that rubisco activation and stomatal conductance were restored. CO_2_ values were then increased stepwise to 500, 650, 800, and 1000 μM with matching and logging performed as before.

The resulting A/Ci curves were fitted using the Excel tool from Zhou et al. [49], which implements the model of Yin and colleagues [50] with the following modifications: changes to the constants found in cells P22, Q22, D126, and D127 (calculations of variables x_1_ and x_2_ for the electron transport limited state) were corrected to match the equations from Yin and colleagues [50], and α (the fraction of O_2_ evolution occurring in the bundle sheath) was assigned the value of 0 as PSII activity is negligible in bundle sheath cells in NADP-ME type monocot C_4_ photosynthesis [51]. The model of Zhou et al. [49] can be used to distinguish not only between limitation by carboxylation reactions vs regeneration reactions, but also between limitation by rubisco vs PEPC [49]. The effect of temperature on each parameter of the equation (*V*_cmax_, *V*_pmax_, g_m_, J, R_d_, K_c_, K_o_, γ*, K_p_, and g_bs_) is accounted for in the Excel tool, which uses an Arrhenius equation to estimate parameter values at measurement temperature, ensuring that the modeled fit is correctly parameterized for the measured temperature [49]. For all A/C_i_ measurements, the first measurement at 400 μM CO_2_ was checked for stomatal limitation according to equation 20 in Long and Bernacchi [48] and any dataset in which stomatal conductance limited photosynthesis by more than 30% was discarded. This initial measurement was also compared with the A/Ci curve fit to determine the limitation to photosynthesis. Representative A/Ci curves and fits can be found in Supplementary Figure S2, and all A/Ci curve fit variables may be seen in Supplementary Table S1.

### Tissue harvest and protein extraction

Leaf tissue from plants subjected to heat in growth chambers was harvested after 0, 1, 6, or 48 h of heat exposure during the middle of the photoperiod, flash-frozen in liquid nitrogen, ground with mortar and pestle and pooled prior to storage at −80°C. All tissue samples contained leaves from at least three plants. Care was taken to exclude the ligule, the midrib, and leaves that were not yet fully expanded or which were senescent. RCA was extracted by ammonium sulfate precipitation according to the protocol of Barta et al. [52] in batches of 100 g with the modification that the 4.1 M ammonium sulfate was adjusted to pH 4.6 instead of 7.0. Purified protein was resuspended to a final volume of 2.5 mL in Leaf Resuspension Buffer (50 mM HEPES-KOH, pH 7.0, 2 mM MgCl_2_, 100 mM KCl, 5 mM D/L-dithiothreitol, 1 mM ATP, 1 mM PMSF, and 10 mM leupeptin). Aliquots were stored at −80°C until use. When compared with samples purified using the complete protocol of Barta and colleagues [52], samples using only the modified ammonium sulfate precipitation method were considerably cleaner and gave similar thermal curve results (Supplementary Figure S3). Tissue was harvested from sorghum at 7 h of heat treatment rather than 6 due to a gun-related lockdown at the ideal time of harvest.

### Western blotting

Crude protein extracts from the first step of purification were resolved by SDS-PAGE on a 12.5% (w/v) gel and transferred onto PVDF membrane using an Invitrogen Power Blotter semidry transfer apparatus according to the manufacturer's instructions. Crude extract (20 μg) was used for maize, setaria, and sorghum, and 5 μg was used for reference species tobacco, Chlamydomonas, Arabidopsis, and spinach. The membrane was blocked with 5% (w/v) nonfat dry milk in PBS supplemented with 0.1% (v/v) Tween-20 (PBST) with shaking at 4°C. Primary antibody (anti-RCA, rabbit, 1:10000 Agrisera AS10 700) was added and incubated for 1 h. The membrane was thoroughly washed with PBST and bound with secondary goat anti-rabbit-HRP (MilliPore) at 1:50000 for 1 h. The membrane was thoroughly washed and visualized with the FemtoGlow Western Plus chemiluminescent diagnostic kit (Michigan Diagnostics) according to the manufacturer's instructions.

### Recombinant protein production

The coding sequence of *Mesembryanthemum crystallinum* thioredoxin F was codon-optimized for expression in *Escherichia coli*, synthesized, and cloned into pET28a with an N-terminal 6xHis tag. Transformed BL21-DE3 was grown to OD_600_ 0.6–0.8, induced with 1mM IPTG and incubated at 12°C overnight. TrxF was purified by Ni-NTA affinity chromatography in buffer containing 50 mM HEPES pH 8.0, 0.5 M NaCl, 5% (v/v) glycerol, 10 mM β-mercaptoethanol, and 5-300 mM imidazole, then dialyzed into the same buffer without imidazole and stored in aliquots at −80°C. TrxF activity was verified using the insulin reduction assay [53].

### Biochemical assays

RCA thermostability was measured with an NADH-linked ATPase enzymatic assay in microtiter plates based on the protocol of Barta et al. [54]. Briefly, 20 μL of purified RCA was aliquoted into 0.2 mL tubes and incubated in a gradient thermocycler for 1 h at temperatures ranging from 30–66°C and returned to ice until measurement, while a control aliquot was maintained on ice. RCA sample (5 μL) was added to 120 μL of reaction mixture in microtiter plates and the change in A_340_ was monitored for 10 min using a Tecan plate reader. ATPase activity was calculated as the initial slope of the line (change in absorbance per minute). T_50_ was calculated by fitting a 4-parameter logistic equation to the dataset using the calculator at (AAT BioQuest Inc, 2022), see example in Supplementary Figure S4. Temperature response data that showed two distinct regions of response (sorghum and tobacco) were divided into two separate curves and each curve was fitted separately to produce two T_50_ values. The two plateaus are unlikely to represent a true response of RCA to heat. Previous work in spinach has shown that mixing of isoforms of different thermal stabilities confers an overall RCA response intermediate between the individual isoforms [33]. Rather, the lower of the two T_50_ values is likely another ATPase present which was not fully removed by our purification technique. The portion of total ATPase activity due to the low-thermal-stability ATPase was calculated and subtracted from total ATPase activity results.

Response of ATPase activity to thioredoxin F, Mg^2+^, and ADP:ATP ratio was measured according to the method of [22,56] because the presence of these small molecules interferes with the NADH-linked assay used to measure T_50_. Briefly, 40 μg of purified enzyme was incubated in 100 mM Tris-HCl pH 8.0 with 5 mM D/L-dithiothreitol with or without 2.6 μg of TrxF as indicated for at least 20 min at room temperature to allow reduction of RCA. ATPase activity was then assayed in a total volume of 50 μL of reaction buffer (50 mM tricine, pH 8, indicated concentration of MgCl_2_, 20 mM KCl, 4 mM total of ATP and ADP according to ratio) for 1 h. SDS and ammonium molybdate reagent were added and allowed to react for 5 min. Color development was terminated by addition of sodium meta-arsenite reagent and A_850nm_ was read. Because the amount of ATP and the presence or absence of TrxF contributed different amounts to the background, no-RCA controls were performed for all treatments and the background was subtracted from all samples prior to statistical analysis. Measurements were normalized to ATP:ADP = 1:0 without TrxF for all time points.

### Statistical analysis

Outliers were removed by Grubbs' test, *P*<0.05. For physiological measurements, significant differences between treatments were determined by Student's T-test with Bonferroni correction. For ATPase assays, a two-way ANOVA with post-hoc Tukey test was used to examine the interaction between ATP:ADP ratio and redox regulation; between heat exposure and ATP:ADP ratio; and between heat exposure and magnesium concentration.

## Results

### Response of CO_2_ assimilation to heat

Sudden exposure to elevated temperatures results in a loss of CO_2_ assimilation capacity. We therefore first sought to obtain CO_2_ assimilation profiles from maize, sorghum, and setaria exposed for 1 h to a range of temperatures between 25°C and 45°C (Supplementary Figure S5A–C). Peak assimilation occurred between 32°C and 35°C for all species. When temperatures exceeded 40°C, all plants displayed reduced capacity to assimilate CO_2_ with sorghum retaining a greater proportion of its peak activity at 42°C than maize B73 or setaria. At 45°C, assimilation had declined to below 20% of the maximum CO_2_ assimilation rate in all plants (Supplementary Figure S5A–C). In line with other studies, we therefore chose 42°C to study the impact of elevated temperatures on CO_2_ assimilation.

**Figure 1:**
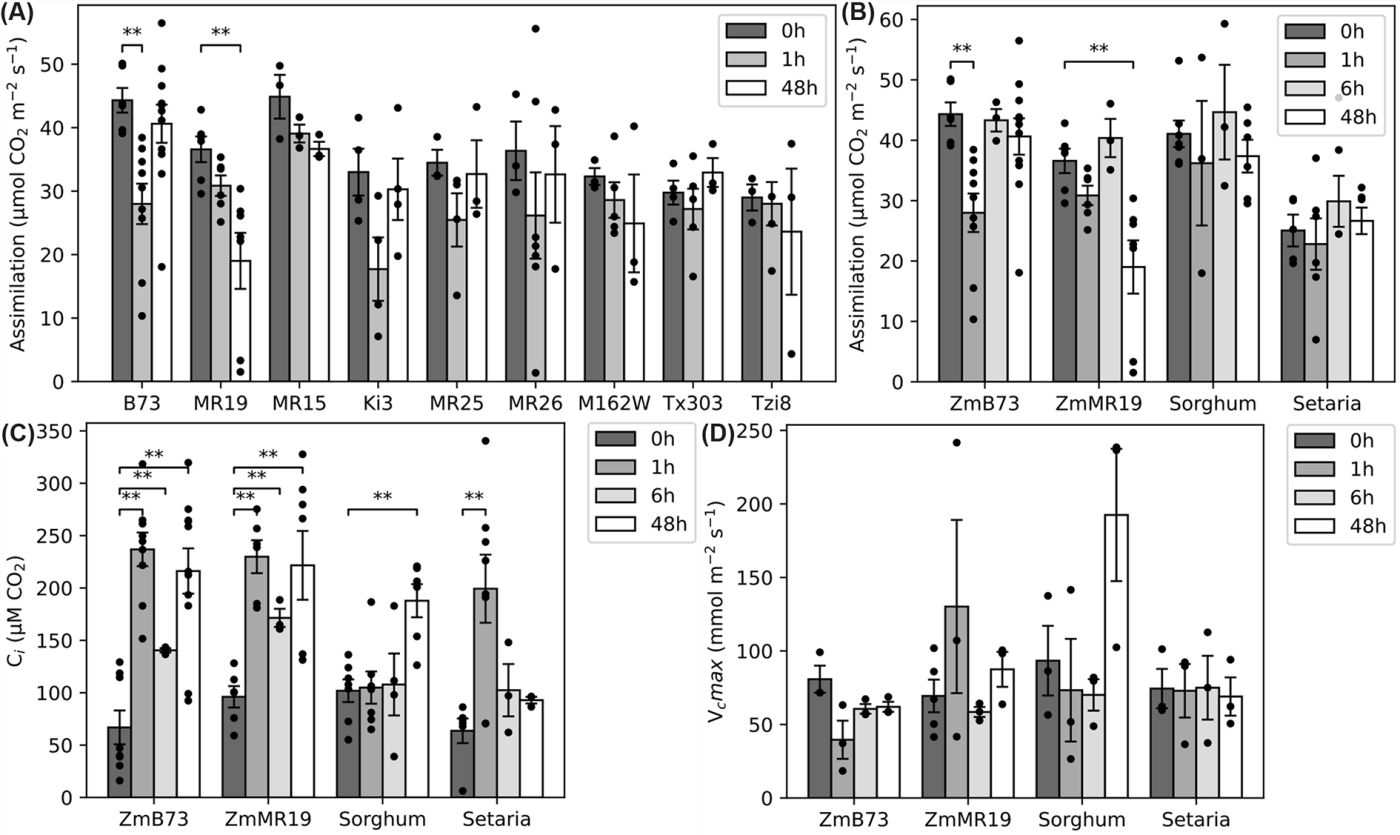
Gas exchange measurements during acclimation to 42°C heat (**A**) Assimilation of various cultivars of maize. (**B**) Assimilation of maize (Zm), sorghum, and setaria. (**C**) C_i_ of maize, sorghum, and setaria at 400 ppm CO_2_. (**D**) V_c_max of maize, sorghum and setaria at 400 ppm CO_2_. *P<0.05, **P<0.01 vs 0-h control by Student's T-test. Individual values are overlaid on the means. Error bars are standard error of at least three biological replicates.

Because there is known variability in RCA expression among maize cultivars [57], we next analyzed the capacity to acclimate the CO_2_ assimilatory capacity to elevated temperature in nine maize cultivars using a time course, starting at 25°C (0 h, prior to treatment), and sampling at 1 h and 48 h after transition to 42°C. Maize B73 showed a significant reduction in CO_2_ assimilation capacity after 1 h but recovered to levels similar to 25°C within 48 h after transition to 42°C. Cultivars MR15, MR25, MR26, and M162W were more resistant to the increase in temperature than B73. We found that three other cultivars shared the B73 response to sudden heat change, Tzi8, KI3, and Tx303. All four cultivars showed the same response, reduction after 1 h, followed by a full recovery by the end of the study (Figure 1A). MR19 retained high CO_2_ assimilation rate after 1 h but displayed a significantly reduced assimilation after 48 h of heat exposure, indicating a failure to acclimate to elevated temperatures. This cultivar was chosen for further study because of its contrasting response to heat compared with B73. Chlorophyll fluorescence data for select maize cultivars is found in Supplementary Figure S6.

We next performed a more in-depth characterization of the heat response of CO_2_ assimilation for maize B73, maize MR19, *Setaria viridis* A10, and *Sorghum bicolor* BTx623. We measured both assimilation and response to CO_2_ (A/Ci) at 25°C (0 h, prior to treatment), 1, 6, and 48 h after transition to 42°C (Figure 1B, Supplementary Figure S2, and Supplementary Table S1). Sorghum and setaria showed consistent CO_2_ assimilation throughout the course of the acclimation experiment.

Intercellular CO_2_ concentration (C_i_) was measured for all species at normal and elevated temperature at 400 ppm CO_2_ (Figure 1C). All species exhibited elevated C_i_ at high temperatures. In both maize cultivars, the C_i_ more than doubled within 1 h and remained elevated. In sorghum, C_i_ remained low until the 48-h time point. In setaria, the increase in C_i_ was transient, and C_i_ returned to pre-treatment values within 6 h. Stomatal conductance in all plants increased during acclimation and paralleled the trends seen in C_i_ (Supplementary Figure S5D).

### Limitations to photosynthesis

Stomatal conductance was not the primary limitation to assimilation at any time point. We calculated stomatal limitation according to equation 20 in Long and Bernacch [48], and only three plants out of the 51 measured exhibited stomatal limitation of more than 30% (Supplementary Table S1). To determine the limitation to CO_2_ assimilation at atmospheric CO_2_ levels (400 ppm), we measured CO_2_ assimilation dependent on C_i_ (A/C_i_ curves) in triplicate for each of the four plants after 0, 1, 6, and 48 h of heat exposure and fit the measurements to a model of C_4_ photosynthesis, as described in detail in [49] (Supplementary Figure S2 and Supplementary Table S1). At ambient temperatures and 400 ppm CO_2_, both maize cultivars were strictly limited by the carboxylation reactions, while one sorghum plant was limited by RuBP regeneration rather than RuBP carboxylation. All setaria cultivars were limited by RuBP carboxylation, but one setaria plant was concurrently limited by PEP regeneration. At the 1 and 6 h time points, the variability among biological replicates was unacceptably high and no conclusion could be reached. At 48 h, setaria and maize remained limited by carboxylation, but all sorghum plants were limited by RuBP regeneration.

### RCA expression and proteoform composition in response to heat

**Figure 2:**
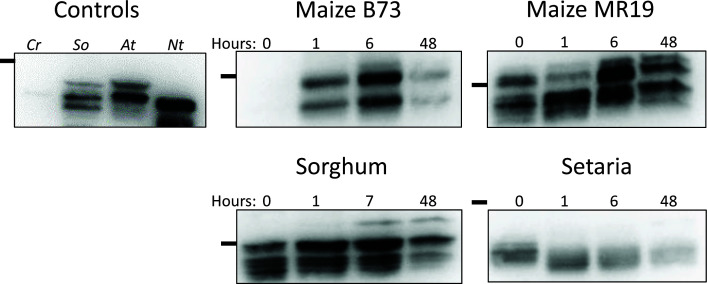
RCA abundance and proteoform distribution during acclimation to 42°C heat Immunodetections were performed on 20 μg of crude protein extract for C_4_ species and 5 μg of crude protein extract for controls. Cr *C. reinhardtii*, So *S. oleracea*, At *A. thaliana*, Nt *N. tabacum*. Times are indicated for each sample in hours. Complete blots may be viewed in the supplement. Black line indicates 37 kDa marker.

Next, we determined the abundance and proteoform composition of RCA in the two maize cultivars, sorghum and setaria. Immunodetections were performed on crude leaf tissue extracts before and after heat treatment, again time-dependently, with RCA-specific antiserum (Figure 2). We first validated our method with RCA from model organisms that had been described previously in the literature (Figure 2). Arabidopsis and spinach samples contain an ⍺ isoform at 45 kDa, β at 43 kDa, and a proteolytic product of β at 41 kDa, with Arabidopsis proteins running slightly higher than their spinach equivalent. Tobacco and Chlamydomonas express only the β isoform, and in our detections both species lack the higher molecular weight bands corresponding to the ⍺ isoform. While Chlamydomonas shows only a single proteoform, tobacco displays several proteoforms of β, including a band at a slightly lower molecular weight of about 39 kDa (Figure 2, see also complete blots in Supplementary Figure S7).

In our C_4_ grasses, RCA abundance increased after transfer to higher temperatures in maize and sorghum, evident already after 1 h of heat treatment and peaking around the 6-h time point. Setaria did not exhibit increased RCA abundance. Maize cultivar B73 showed much lower expression of RCA at ambient temperatures compared to after heat exposure. For all plants, we found the expression of at least 3 RCA proteoforms. In both maize cultivars and in sorghum, we found an additional higher molecular weight band that appeared only after heat treatment, most prominently at 6 h. Other changes in proteoform abundance were specific to the species or cultivar. In setaria the larger proteoform is depleted within 1 h of heat treatment and does not recover during the observation period, but the smallest proteoform is transiently enriched in response to heat. In maize MR19 there is a transient decrease in the large isoform after 1 h of heat exposure. Sorghum shows the least amount of change in protein composition overall outside of the appearance of the largest product.

### RCA ATPase activity in C_4_ plants at elevated temperatures

**Figure 3:**
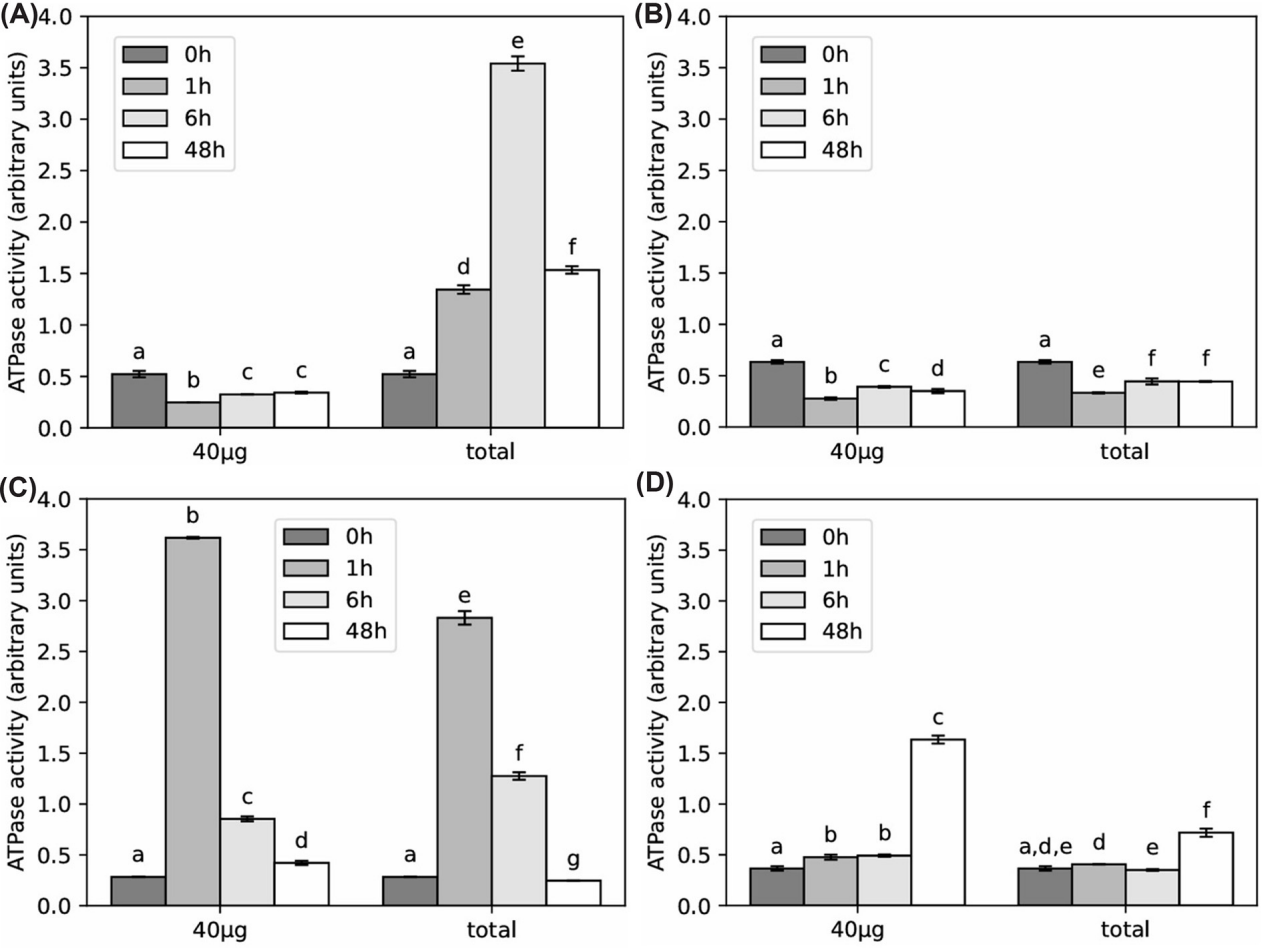
ATPase activity of RCA (**A**) maize B73. (**B**) Maize MR19. (**C**) Sorghum. (**D**) Setaria. Letters denote significance within data series of *P*<0.01 by ANOVA with post-hoc test. Error bars are standard deviation of n = 5.

We next characterized RCA's ATPase activity in different settings relevant for heat acclimation. Recombinantly produced proteins are commonly used for biochemical assays, but due to the complexity of RCA proteoforms and importance of post-translational modifications, we purified RCA directly from plant tissue. First, we examined how RCA's ATPase activity is affected by exposure to elevated temperatures. We normalized the ATPase activity either per μg isolated protein (black bars), to capture the intrinsic activity of RCA itself, which can change due to modifications and proteoform composition changing upon heat exposure, or to total leaf content by mass of plant tissue by using equal volumes of protein extract (white bars), allowing to assess the total capacity of RCA for rubisco reactivation, including adjustments made to total RCA protein content. In maize B73, total RCA activity increased substantially, by ∼7-fold, at 42°C, peaking at 6 h after heat exposure (Figure 3A). This increase was solely driven by the increase in RCA protein abundance (Figure 2) because intrinsic RCA activity (per μg protein) was reduced immediately after heat exposure and only recovered slightly by 48 h. In maize cultivar MR19, which fails to acclimate to heat (Figure 1), total RCA activity and intrinsic activity are closely linked and were reduced dramatically directly after exposure to elevated temperatures (1 h time point, Figure 3B). RCA activity subsequently only recovered slightly, resulting in only ∼70% of ambient total RCA activity retained at 42°C in a similar profile as maize B73 (Figure 3A,B). Both sorghum and setaria showed a close connection of intrinsic and total RCA ATPase activity, but, while sorghum peaked at 1 h, it took setaria longer to increase its RCA activity. By the end of the experiment, intrinsic RCA activity was increased 5-fold in setaria, while ATPase activity in sorghum was fully attenuated back to initial levels (Figure 3C,D). Taken together, RCA ATPase activity in maize was reduced early after exposure to elevated temperatures, which appears to be compensated by increasing overall RCA abundance in thermotolerant cultivars. Sorghum and setaria both manage to increase intrinsic ATPase activity in response to heat to maintain their CO_2_ fixation capacity but on very different timelines.

### RCA thermostability

**Figure 4:**
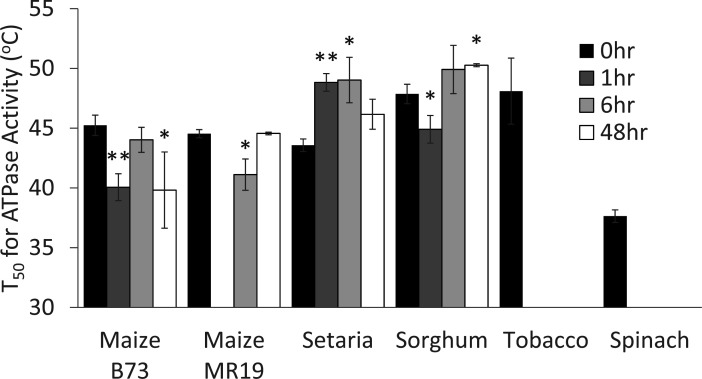
Temperature sensitivity of ATPase activity *P<0.05, **P<0.01 relative to 0 h by Student's T-test, error bars are standard deviation of n = 3 technical replicates.

Our data indicate that different plant species (and even different cultivars of the same species) show flexibility in how RCA abundance and activity change during heat acclimation. We next sought to determine how these changes affect RCA thermostability. We measured RCA thermostability by pre-incubating purified enzyme for 1 h at temperatures ranging from 30°C to 66°C, then measuring activity using a spectroscopic ATPase assay based on NADH consumption (Figure 4). The resulting heat-response curves were fitted to determine the temperature at which half of the activity was lost (T_50_). Spinach RCA had a lower T_50_ than tobacco RCA (37.6°C vs 48.0°C). The thermostability of RCA extracted from both maize cultivars varied with time but never increased beyond the 0-h control. In contrast, both setaria and sorghum RCA displayed increased T_50_ at various times, with setaria increasing from 43.6°C (0 h) to 49.0°C (6 h), and sorghum increasing from 47.9°C (0 h) to 50.3°C (48 h).

### Effect of ATP/ADP ratio on RCA ATPase activity

**Figure 5:**
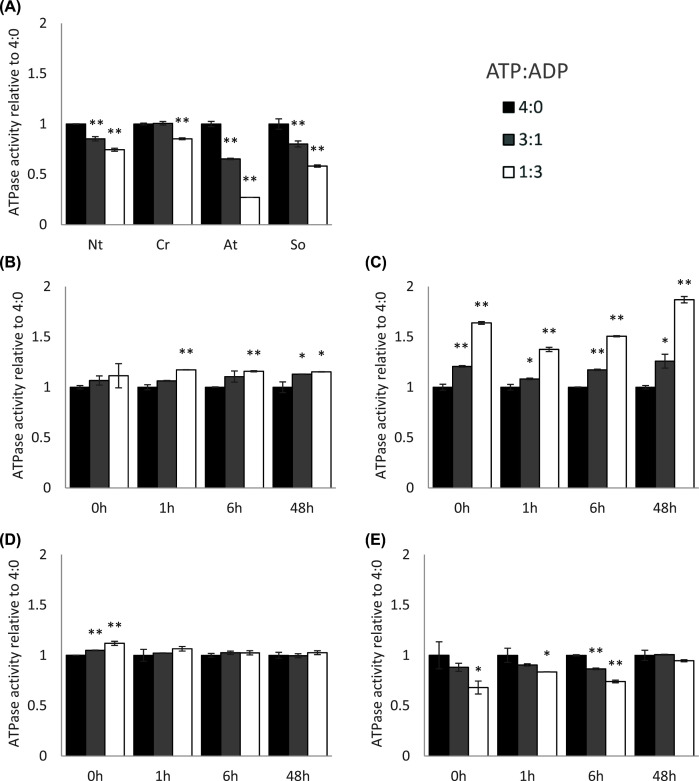
Response of ATPase activity to changes in ATP:ADP ratio. All measurements performed with 10 mM Mg^2+^ (**A**) Controls not treated with heat. (**B**) Maize B73. (**C**) Maize MR19. (**D**) Sorghum. (**E**) Setaria. *P<0.05, **P<0.01 relative to 4:0 ratio by ANOVA with post-hoc Tukey test, error bars are standard deviation of n = 5.

Previous work has established that the ATPase activity of RCA is inhibited by increasing concentrations of ADP [18]. We replicated the ADP-dependent inhibition of RCA's ATPase activity with RCA purified from Arabidopsis, spinach, tobacco, and Chlamydomonas (Figure 5A). Using a two-way ANOVA, we found statistically significant differences in ATPase activity in response to ADP concentration in all four plants, albeit the significant differences occurred at different time points. While maize B73 was not affected by the ATP:ADP ratio at all at ambient temperature (Figure 5B), sorghum and maize MR19 showed increased ATPase activity with a higher ADP:ATP ratio (Figure 5C,D). At ambient temperatures, ATPase activity in RCA from setaria was similarly inhibited by ADP (Figure 5E). In response to elevated temperatures, maize B73 showed slight but significant stimulation by increased levels of ADP (Figure 5B). Maize MR19 was stimulated by ADP at all times (Figure 5C). Sorghum RCA lost its sensitivity to ADP immediately upon heat exposure (Figure 5D). Setaria also lost ADP sensitivity, but only after 48 h of heat (Figure 5E). Taken together, setaria plants were the only ones in our set of C_4_ plants that showed classic ADP-dependent inhibition of ATPase activity, all other species showed some amount of stimulation by increased ADP amounts. The interaction between heat exposure time and ADP was significant in both setaria and maize MR19 (*P*<0.001), but not sorghum or maize B73 (*P*>0.05).

### Effect of Mg^2+^ on RCA ATPase activity

**Figure 6:**
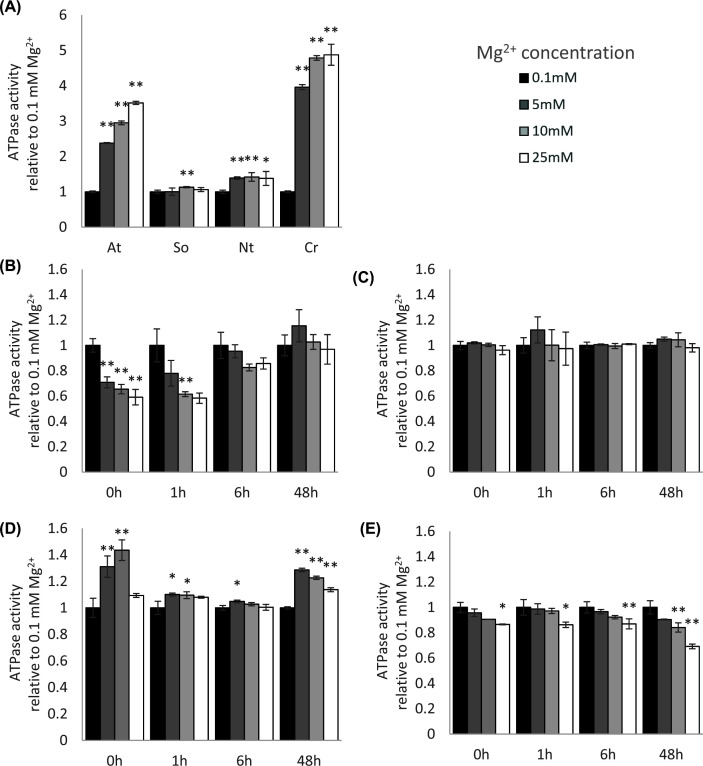
Response of ATPase activity to changes in Mg^2+^ concentration. All measurements performed with ATP:ADP = 4:0 (**A**) Controls not treated with heat. (**B**) Maize B73. (**C**) Maize MR19. (**D**) Sorghum. (**E**) Setaria. *P<0.05, **P<0.01 relative to 0.1 mM Mg^2+^ by ANOVA with post-hoc Tukey test, error bars are standard deviation of n = 5.

The availability of magnesium can affect the activity of many enzymes, including RCA. We therefore studied the responsiveness of RCA's ATPase activity to changes in Mg^2+^ concentration. Magnesium concentration affected ATPase activity significantly in maize B73, setaria and sorghum (*P*<0.001), but only marginally in maize MR19 (*P* = 0.018) (Figure 6). Most RCA samples were significantly more active with more Mg^2+^ in the buffer (Figure 6). These increases are consistent with what had been previously reported for tobacco RCA [32]. Of the C_4_ plants analyzed here, only sorghum RCA was stimulated by Mg^2+^ by 43% at 10 mM Mg^2+^ (Figure 6D). Surprisingly, both maize B73 and setaria showed inhibition by increased Mg^2+^ concentrations (Figure 6B,E). Maize RCA was reduced to 34% activity by 10 mM Mg^2+^ (*P*<0.01), while setaria showed less of an effect. Additionally, the interaction between heat exposure time and magnesium concentration was significant in maize B73 and sorghum (*P*<0.001) and in setaria (*P* = 0.0014) by two-way ANOVA. In sorghum, RCA responsiveness to Mg^2+^ was transiently dampened, to below <10% increase at 1 and 7 h of heat treatment. B73 RCA, which was most strongly inhibited by Mg^2+^ at ambient temperatures, lost the Mg^2+^-dependent inhibition gradually after heat exposure. For setaria, the inhibition was less pronounced (9% at 10 mM Mg^2+^, not significant) at ambient temperatures and only rose to the level of significance after 48 h of heat treatment (16% inhibition at 10 mM Mg^2+^, *P*<0.01).

### Redox sensitivity

TrxF-mediated redox regulation of the disulfide bond in the C-terminal domain of the α isoform of RCA is known to be important in C_3_ plants [18,58]. However, in NADP-malic enzyme (NADP-ME)-type C_4_ plants such as maize, sorghum, and setaria, NADPH reducing equivalents are imported into bundle sheath cells from mesophyll cells, and electron transport in bundle sheath cells (BSCs) is therefore specialized for ATP production via cyclic electron flow rather than for producing reducing equivalents. The redox state of BSCs has not been well characterized, and the extent of TrxF-mediated redox regulation is currently unknown. Nevertheless, we examined this response in our C_4_ plants. Maize RCA was slightly stimulated by TrxF-mediated reduction, and sorghum and setaria were insensitive (Supplementary Figure S8).

## Discussion

Analyzing gas exchange measurements, enzymatic activities, protein abundance, and proteoform composition allowed us to compare the properties of RCA proteins across C_4_ monocot plants, which helps our understanding of the acclimation strategies that these plants employ to maintain CO_2_ assimilation at elevated temperatures. We found that in maize and setaria, similar to earlier reports, CO_2_ assimilation at high temperature and ambient CO_2_ was limited by carboxylation [40,59–62]. Limitation by RuBP carboxylation is consistent with RCA failing to maintain rubisco in a catalytically competent state. Heat-induced changes in RCA expression and proteoform composition appear to be critical components of heat acclimation in maize and sorghum, but are not involved in heat acclimation in setaria. The RCA activity of all C_4_ species studied was affected by varying concentrations of ADP and Mg^2+^. However, there were substantial differences in the way these crops employed the various levels of RCA regulation to maintain photosynthetic carbon fixation.

### Species-specific strategies for high temperature acclimation

Setaria, maize, and sorghum assimilated CO_2_ efficiently over a range of temperatures up to 40°C, where we first found CO_2_ assimilation rates starting to decline. At 42°C heat, all plants maintained their carbon fixation capacity after 48 h of exposure, with compensatory changes apparent both at the physiological level (e.g., stomatal opening) and molecular level (e.g., protein abundance and proteoform composition). Notably, maize cultivar MR19 failed to acclimate to high temperature despite physiological and molecular changes and did not maintain its initial CO_2_ assimilation rate after 48 h. We observed altered RCA abundance and proteoform composition during heat acclimation. These results are broadly similar to those reported by Kim et al., and specific differences in the extent and timing of changes are likely attributable to differences in the genotypes and antibodies used for the immunoblot assays [15]. However, RCA abundance alone has not proven predictive of its capacity to maintain rubisco activity [20]. Our results corroborate with this observation, as in both MR19 and sorghum RCA abundance was not well correlated with assimilation. This suggests that additional factors such as regulation affect the activity of RCA, such as local environmental stimuli (e.g., temperature, Mg^2+^, and ADP/ATP ratio). However, the timing, magnitude, and direction of all these changes differed substantially between the different species.

#### Maize

There was a wide range of CO_2_ assimilation responses observed at 42°C across multiple maize cultivars. While several maize cultivars showed no decline in CO_2_ assimilation, even early during heat acclimation, maize B73 transiently decreased CO_2_ assimilation and MR19 failed to acclimate after extended exposure to heat. In both cultivars, rapid physiological changes occurred within the first hour of heat exposure: increases in stomatal conductance and a corresponding increase in C_i_. Both cultivars remained limited by RuBP carboxylation, and there was no change to *V*_cmax_ throughout the duration of heat treatment (Supplementary Table S1). During acclimation, there were dynamic biochemical changes in B73: a rapid but transient increase in RCA abundance, appearance of a higher molecular weight proteoform of α, rapid gain of stimulation by ADP, gradual loss of inhibition by Mg^2+^, and a slow increase in q_N_ (Supplementary Figure S6). Both maize cultivars fail to increase their intrinsic RCA ATPase activity or thermostability (T_50_) in response to heat, and even showed reduced ATPase activity after heat exposure. B73 compensates by increasing the abundance of RCA, managing an overall increase in RCA ATPase activity, which does not occur in MR19. In contrast, the biochemistry of MR19 was similar to the B73 acclimation endpoint at all time-points. Even at the 25°C 0-h measurement, MR19 displayed high RCA abundance, ADP sensitivity, Mg^2+^ insensitivity, and high q_N_. The appearance of the higher molecular weight proteoform was a transient event in B73, while it can be observed in MR19 at all time points, even at ambient temperatures. It appears that even at the greenhouse condition (28°C), MR19 constitutively activates the biochemical program of RCA heat acclimation but cannot sufficiently elevate its RCA activity when further challenged at 42°C heat. Some cultivar-specific genetic determinants of RCA expression are known [57], but the interactions of these polymorphisms with specific environmental conditions has not yet been examined.

#### Sorghum

In sorghum, stomatal conductance and C_i_ changed only after prolonged heat treatment; however, rapid biochemical changes occurred. An immediate, transient increase in RCA abundance was accompanied by a suppression of stimulation by both ADP and Mg^2+^. After 7 h of heat treatment, the higher molecular weight α proteoform appeared and remained even when overall RCA abundance decreased at 48 h. The occurrence of the higher larger RCA coincided with an increase in thermostability, which was not observed in maize. Mg^2+^ sensitivity, but not ADP sensitivity, returned to initial levels by 48 h. Unique to sorghum out of all plants examined was that, by the end of acclimation, RuBP carboxylation was no longer limiting for CO_2_ assimilation. It is noteworthy that in both maize and sorghum, although the increased abundance of RCA was transient, the changes in biochemical properties were sustained. This suggests that these biochemical properties derive from the altered composition of RCA proteoforms, such as might be produced by post-translational modifications. Further study into the exact identities and properties of these proteoforms should be pursued to more fully understand this stress response.

#### Setaria

While maize and sorghum both exhibit an immediate increase in RCA abundance and rapid changes to RCA's enzymatic properties, setaria RCA protein abundance never increased and enzymatic changes took at least 6 h to manifest. Instead, setaria appears to execute an immediate but transient C_i_ increase mediated by marginal changes in stomatal conductance. Although the abundance of RCA appears unchanged throughout the time course, the proteoform composition of the lower molecular weight bands is shifted early, coinciding with a small but significant, temporary increase in RCA T_50_. At 48 h, setaria displayed elevated per-molecule ATPase activity, loss of ADP inhibition and slight increase in Mg^2+^ sensitivity. Unique among the C_4_ species studied, setaria is ADP-inhibited, matching the profiles of C_3_ species.

### Small molecule regulation may control oligomeric state

The biochemical composition of the chloroplast stroma is heavily influenced by the environment, with changes to redox state, ADP:ATP ratio and thylakoid leakiness known to occur in response to heat [63]. The ATP:ADP ratio is a good indicator of the energy status of the chloroplast: a high ATP:ADP ratio is usually seen in plants with a highly active photosynthetic electron transfer chain, while high amounts of ADP indicate an inadequate supply of ATP for chloroplast energy-dependent metabolic reactions, most prominently carbon fixation via rubisco. The statistically significant interaction between heat exposure duration and ADP and Mg^2+^concentrations suggests plants modulate the sensitivity of RCA activity to these small molecules in response to temperature exposure.

The effects of the biochemical environment on RCA activity and oligomeric state are already known to vary among plant species [27,29,30]. We likewise saw that the responses of C_4_ species differed from those of control non-C_4_ species. For example, in C_3_ plants and algae, Mg^2+^ stimulated ATPase activity in a concentration-dependent manner, but inhibited activity in both maize and setaria. Similarly, high ADP:ATP environments were inhibitory to control plant RCAs but stimulated those from maize and sorghum. We suggest that these results point to differences in the oligomerization of C_4_ RCA.

**Figure 7:**
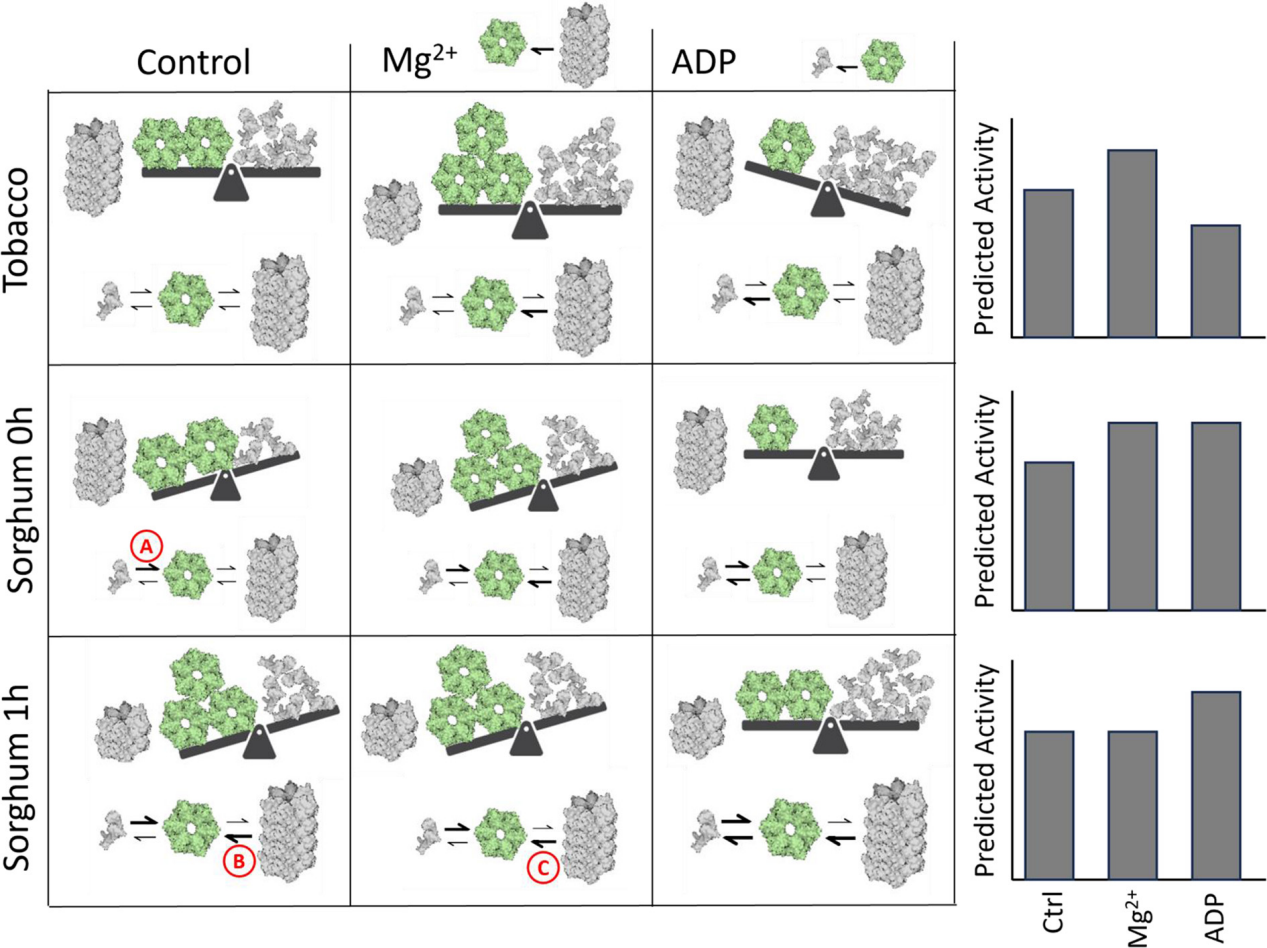
Proposed mechanism for heat-mediated changes in RCA ATPase activity and small molecule responses Imbalance between dimers and hexamers always reduces activity. (**A**) Increased hexamer affinity in sorghum RCA compared to tobacco (above). (**B**) After one hour of heat, subunit aggregation affinities are reduced. (**C**) Addition of magnesium cannot further reduce aggregation affinity at 1 hour of heat. See further description in discussion.

Experimental evidence has resulted in a model of three oligomeric states that coexist within the chloroplast stroma [32]. Inactive dimers assemble into active hexamers, which can form higher-order inactive aggregates. There is an optimal equilibrium ratio between hexamer and dimer states which produces the highest activity, and deviations from that optimum ratio reduce RCA activity. Mg^2+^ promotes disassembly of aggregates and formation of hexamers. ADP promotes the formation of dimers and, at high RCA concentrations, aggregation. It is possible that the ADP-insensitive Rca2β isoform of wheat is more thermosensitive than the ADP-insensitive Rca1 β isoform because its inability to respond to ADP impairs its ability to maintain the optimal oligomeric ratio [24,36,64]. In our preparations of C_3_ plants, Mg^2+^ increases the ATPase activity of RCA by liberating subunits from aggregates and increasing the pool of actively cycling RCA, while ADP decreases activity by shifting the hexamer-dimer equilibrium toward a suboptimal, dimer-dominated state (Figure 7, top row). Kuriata et al. demonstrated that the affinities for hexamer formation and for aggregation are at least partially independent from each other insofar as ATP, ADP, and Mg^2+^ produced different effects on each [27]. It has also been shown that in a mix of proteoforms, the properties of each proteoform contribute to determine the oligomerization state of the entire pool [18,19]. We hypothesize that the changes in proteoform composition seen after heat treatment seen by immunoblot (Figure 2) cause a shift in the relative affinities for hexamer formation vs aggregation. In addition to changes in expression of the α and β isoforms, the bands likely correspond to various post-translational modifications of RCA. For example, a proteolytic product of the β isoform has been described in maize, and the Arabidopsis α isoform is known to be phosphorylated at threonine 78 [9,10,65]. Additional post-translational modifications have been observed but their influence on protein activity is not fully understood [66].

Imagine that the hexamer formation affinity in sorghum RCA is higher than that of tobacco (Figure 7, arrow labeled A). Thus, at the 0-h timepoint, subunits were primarily in the aggregated state and in the hexamer state. Addition of Mg^2+^ would stimulate activity by releasing more subunits from aggregates to participate in the dimer-hexamer cycle, while addition of ADP would increase activity by rebalancing the cycle toward the underrepresented dimer state (Figure 7, second row). After 1 h of heat, changes in proteoform composition could reduce the subunit aggregation affinities (Figure 7, arrow labeled B). Because proteoforms interact to determine the overall phenotype, changes to a small proportion of subunits could destabilize all aggregates, shifting a large proportion of subunits from aggregates into the actively cycling pool. In this way, for example, a loss of Mg^2+^ sensitivity by sorghum isoforms at 1 h (Figure 5) could indicate that the proteoform affinity has changed such that higher-order aggregates have already been destabilized and more subunits cannot be liberated into the active pool by adding Mg^2+^ (Figure 7, arrow labeled C). When a larger proportion of subunits is present in the actively cycling pool than in the inactive aggregate, the activity per unit protein would increase, as observed in Figure 3. Similar explanations based on subunit affinities can be made for the activities of maize and setaria, and experiments like those performed by Kuriata et al. should be used to quantitatively test this proposed mechanism [27].

## Conclusions

Heat acclimation is a dynamic process in which plants modulate their stromal environment and RCA proteoform abundance, accompanied by physiological changes like stomatal conductance that in turn affect intercellular CO_2_ concentration. All these parameters must be considered to obtain a holistic understanding of the mechanisms contributing to thermal limitation in each plant at each time point.

One example is the observation that increased RCA activity in setaria and sorghum is not fully explained by increased RCA abundance. This implies that while overexpressing RCA may improve heat tolerant carbon assimilation in some plant species [67], plants also evolved mechanisms for promoting RCA activity without overexpression, some of which are showcased in this work. Furthermore, the thermostability of the enzyme itself is not predictive of its activity, so attempts to engineer a more thermostable protein may not improve activity *in planta*. Rather, we have shown that RCA activity is modulated in response to heat by a combination of changes in abundance, post-translational modifications and small molecules regulators, all of which likely affect the oligomerization state of RCA. The magnitude and direction of these effects is species-specific and dependent on the duration of the heat treatment. Thus, we caution that observations regarding the heat acclimation strategy in the model species setaria cannot reliably be transferred and used to bioengineer other crop plants. Nor can the effects of the biochemical environment be safely extrapolated even among cultivars of the same species. Future work both on RCA and on modeling C_4_ photosynthesis must therefore be specific to the species and cultivar, as advocated by others [60].

Others have demonstrated the importance of small molecules on determining the oligomerization state of ATPase and its activity using *in vitro* assays [12,19,27–33,64]; however, previous work had not compared multiple species or multiple environmental conditions simultaneously. Although environmentally-induced changes in proteoform composition have been described [11,13–17], the effects of altered proteoform composition on responses to biochemical regulation is also not well understood. This work extends the current knowledge to demonstrate that environmental perturbations that alter the proteoform composition of RCA (such as heat) also alter its responses to small molecules, likely through altered oligomerization properties. We show that the interaction of multiple known mechanisms (protein abundance, post-translational modifications, ADP regulation and Mg^2+^ regulation) contribute to the overall stability and activity of RCA. We suggest that future studies should focus on determining the identities of heat tolerant RCA proteoforms that appear during plant heat acclimation, and on potential changes in abundance of hexamers and aggregates during heat acclimation. It is clear that *in vitro* assays using recombinant protein will not be able to fully capture the biochemical complexity of the chloroplast stroma as well as the proteoform diversity that we observed. A more mechanistic understanding of the molecular events during heat acclimation *in vivo* will likely generate actionable predictions that could improve assimilation in these plants during heat exposure.

## Supplementary Material

Supplementary Figures S1-S8 and Table S1

## Data Availability

All data are contained within this paper and its supporting material, and raw data files are available upon request to the corresponding author at stainbr1@msu.edu.

## Abbreviations

ANOVA: Analysis of variance
HRP: horseradish peroxidase
IPTG: Isopropyl β-D-1-thiogalactopyranoside
PEPC: Phosphoenolpyruvate carboxylase
PSII: photosystem II
PVDF: Polyvinylidene fluoride
