# Supplementary material for: C4 grasses employ distinct strategies to acclimate rubisco activase to heat stress
Source: Biosci Rep. 2024 Oct 23;44(10):BSR20240353. doi: 10.1042/BSR20240353 (PMC11499382; doi:10.1042/BSR20240353)
Supplement: Supplementary Figures S1-S8 and Table S1 [file BSR-2024-0353_supp.pdf]

Supplementary Material to: C_4 Grasses Employ Various Strategies to Acclimate Rubisco Activase to Heat Stress

Sarah C Stainbrook*[1,3], Lindsey N Aubuchon[1], Amanda Chen[2], Emily Johnson[2], Audrey Si[2], Laila Walton[2], Angela Ahrendt[2], Daniela Strenkert[3], Joseph Jez[1]

*** Correspondence:**
Sarah Stainbrook: stainbr1@msu.edu

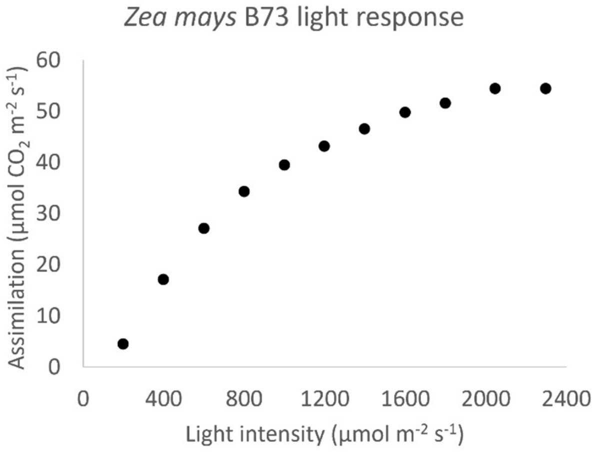

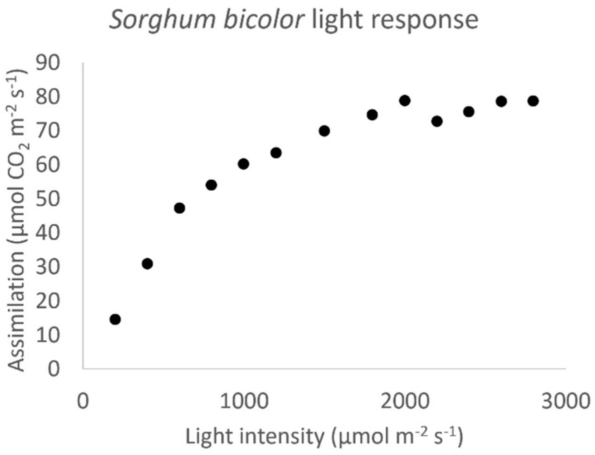

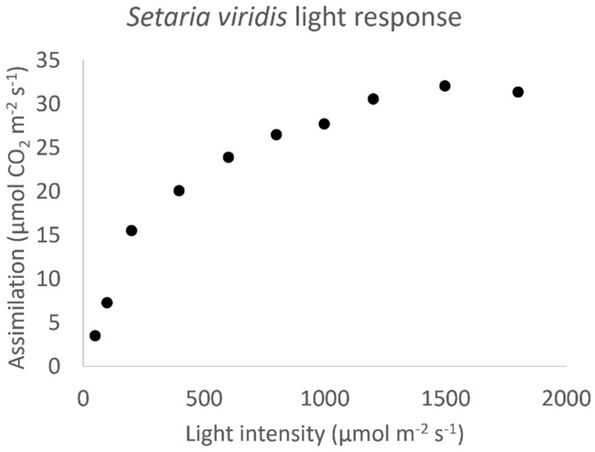

Figure S1: representative light response curves. Responses were measured at 25°C and 400µM CO_2.

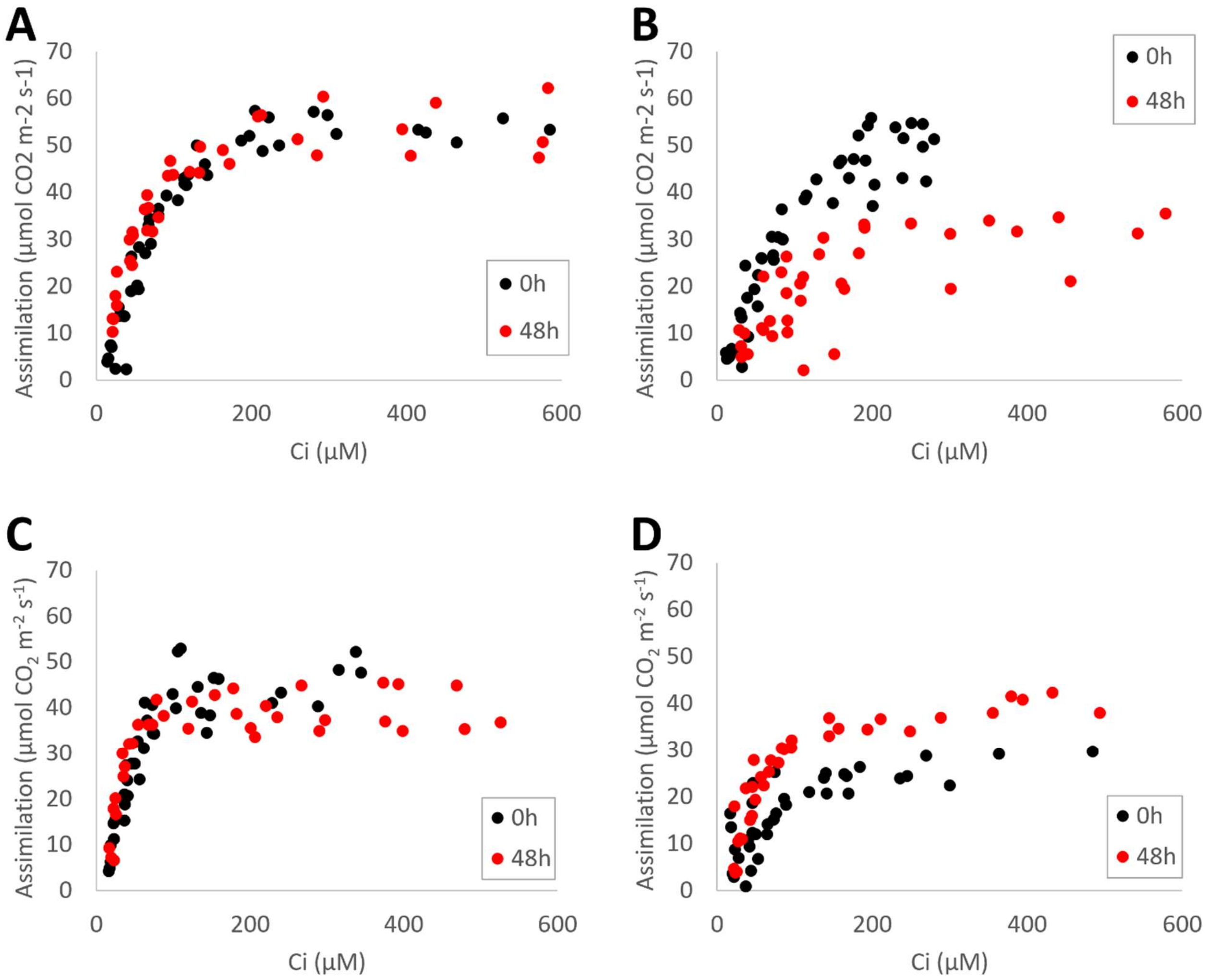

Figure S2: A/Ci curves. Points are individual measurements. **A.** Maize B73. **B.** Maize MR19. **C.** Sorghum. **D.** Setaria

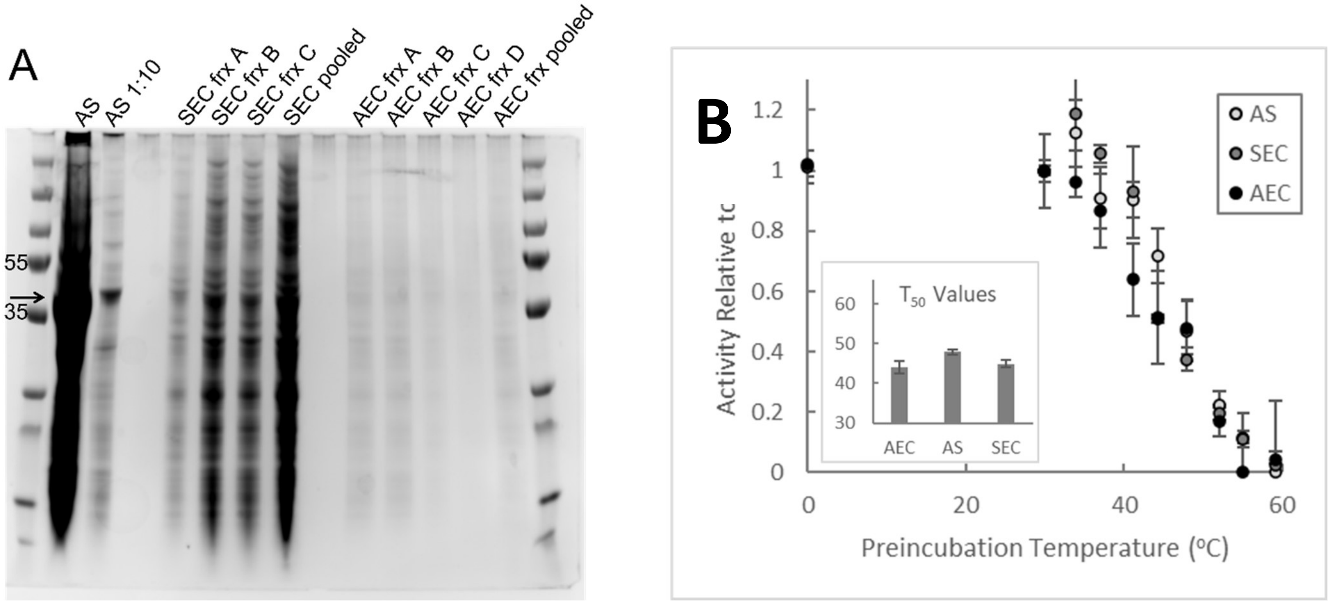

Figure S3: Comparison of RCA purification methods. A. total protein stain for maize tissue purified with ammonium sulfate precipitation only (AS); ammonium sulfate precipitation and size exchange chromatography (SEC); or ammonium sulfate precipitation, size exchange chromatography and anion exchange chromatography (AEC), multiple fractions (frx) shown for SEC and AEC samples. Expected RCA size indicated by arrow. **B.** Thermostability assay on AS, SEC and AEC samples, with calculated T_{50} values in inset. Mean and standard deviation of 3 technical replicates. Differences in T_{50} were not significant.

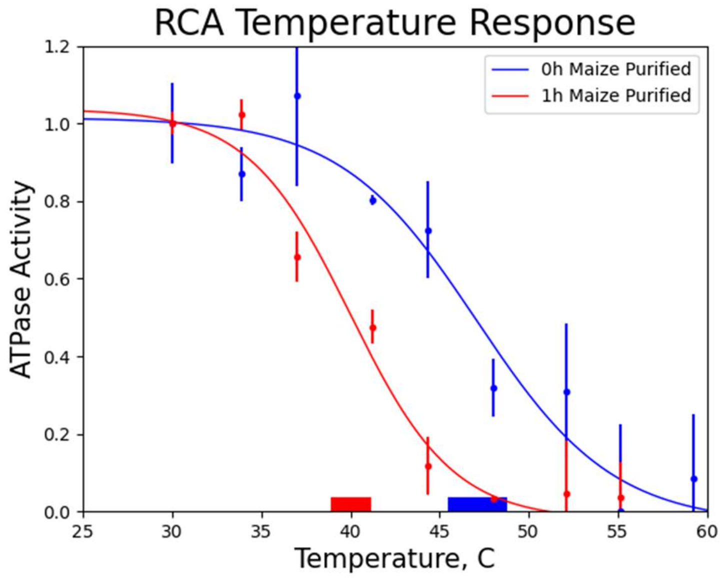

Figure S4: Temperature response curves for maize B73 0h and 1h samples. T_{50} values are represented by bars at the bottom of the graph. Error bars are standard deviation, n=3.

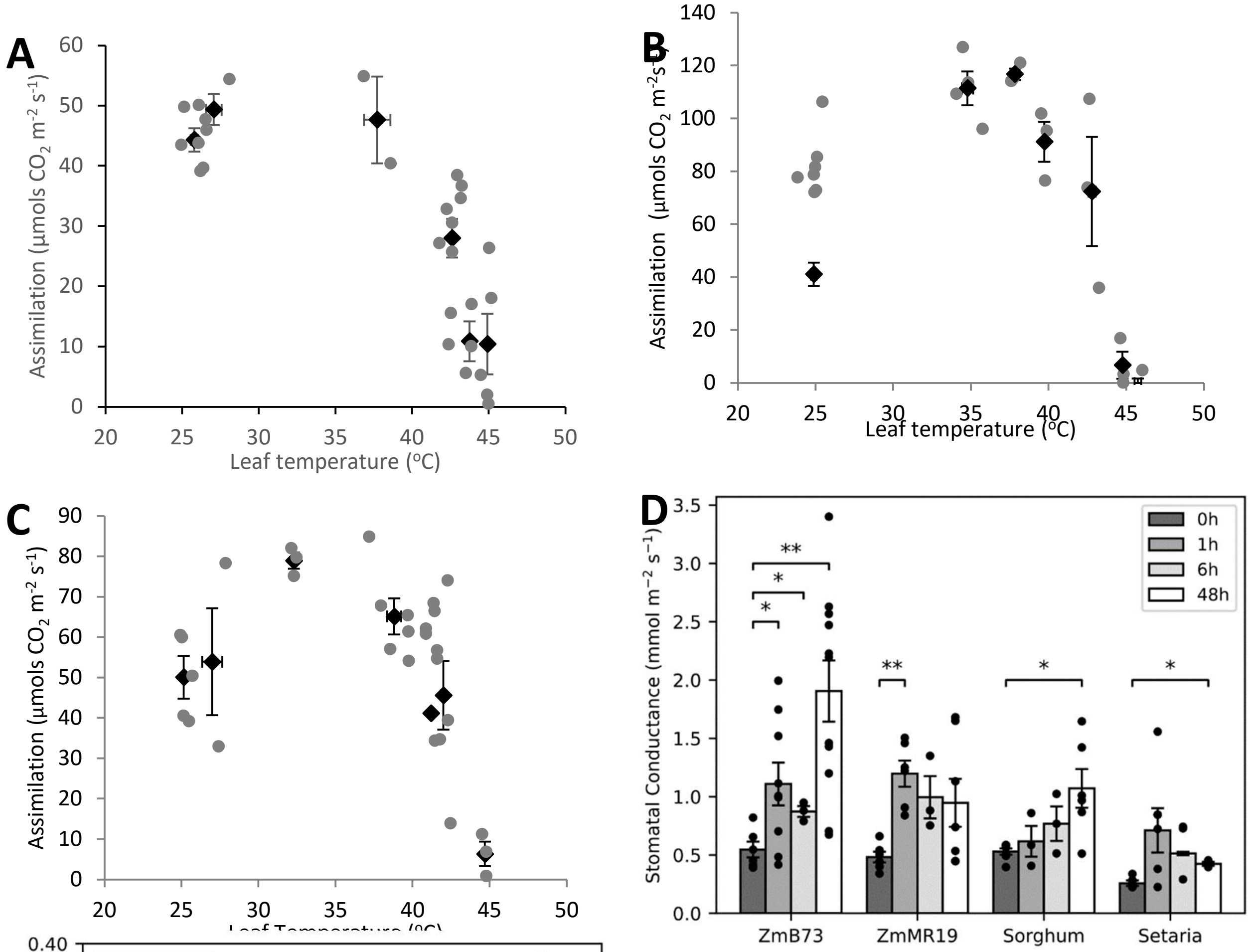

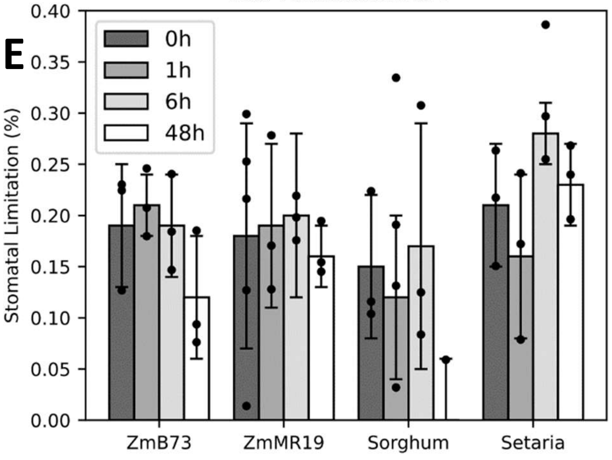

Figure S5: Additional gas exchange data. **A-C.** assimilation after 1 hour treatment at various temperatures. **A.** Maize B73. **B.** Sorghum. **C.** Setaria. Grey points are individual plants, dark points and error bars are means. **D.** Stomatal conductance during acclimation to 42°C heat. **E.** Stomatal limitation of the initial (400µM CO_2) point of all A/Ci curves. * p<0.05, ** p<0.01 vs. 0-hour control by Student's T Test. Error bars are standard error of at least 3 biological replicates.

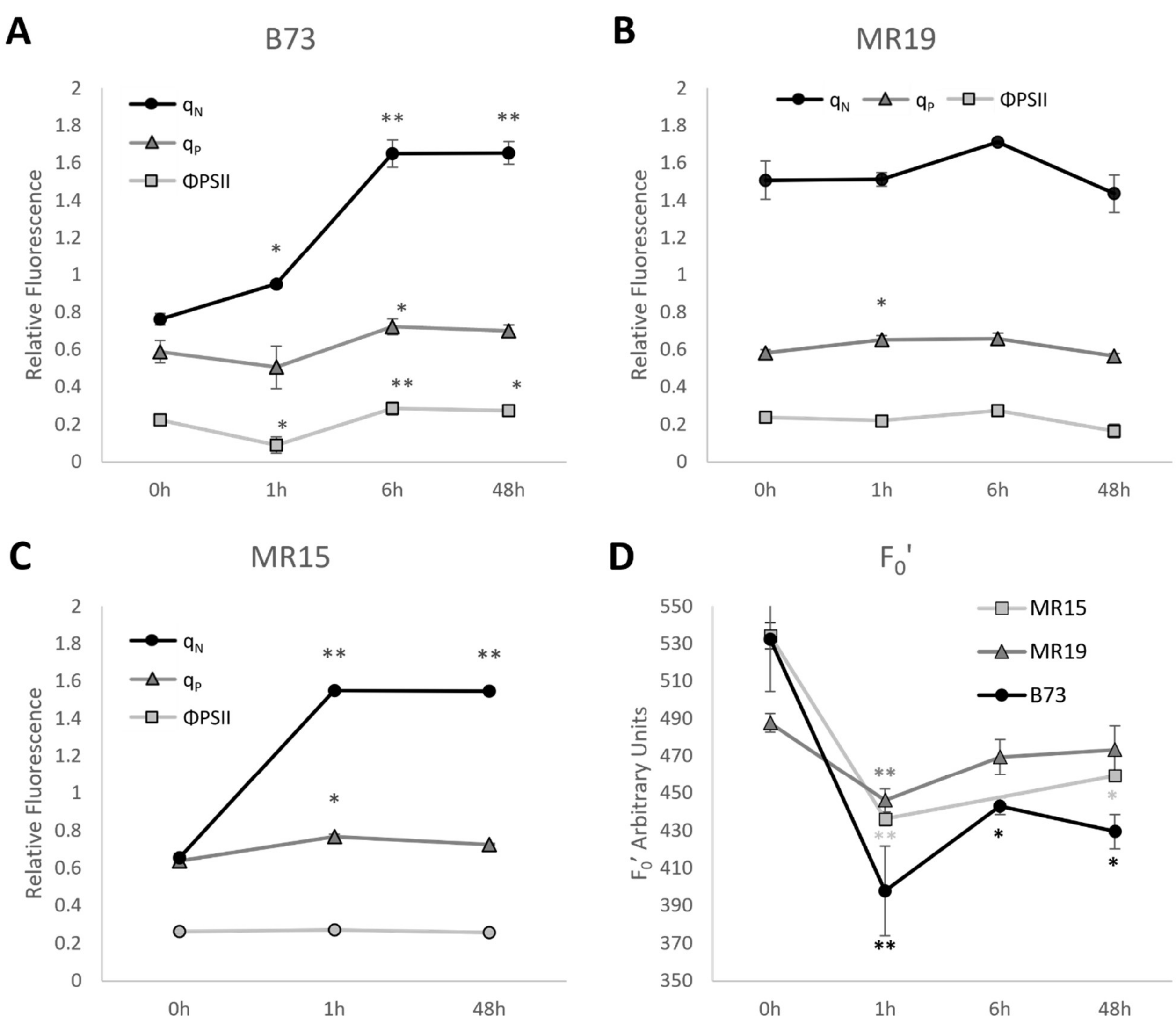

Figure S6: Chlorophyll fluorescence data for select maize cultivars. A-C. Photon partitioning for maize B73 (A), MR19 (B) and MR15 (C). q_N nonphotochemical quenching parameter, q_P photochemical quenching parameter, ΦPSII quantum yield of photosystem II. **D.** Light-adapted basal fluorescence parameter (F_0’) of all three cultivars. * $p<0.05$, ** $p<0.01$ vs. 0-hour control by Student’s T-Test. Error bars are standard error of at least 3 biological replicates.

MR15 (Figure S3C) displays the typical result of heat exposure, with q_N rapidly increasing and remaining elevated throughout the duration of treatment. q_P and ΦPSII remain largely unchanged. In contrast, B73 has delayed induction of q_N, which remains fairly low at 1 hour (Figure S3A). This change is correlated with a transient decrease in ΦPSII. q_N is fully induced by 6 hours and remains elevated at 48 hours, while ΦPSII is slightly higher than the control value at both time points. Interestingly, MR19 displays elevated q_N even at the 0h condition of 25°C (Figure S3B). No changes to q_N, q_P or ΦPSII are seen in MR19 during the heat treatment.

Damage caused by high temperature generally results in an increase in F_0’, while acceptor-side limitations such as CO_2 assimilation result in decreased F_0’ (Sharkey et al., 2001). All three cultivars show significantly reduced F_0’ at 1 hour of heat, indicating that CO_2 assimilation is limiting at that time point (Figure S3D).

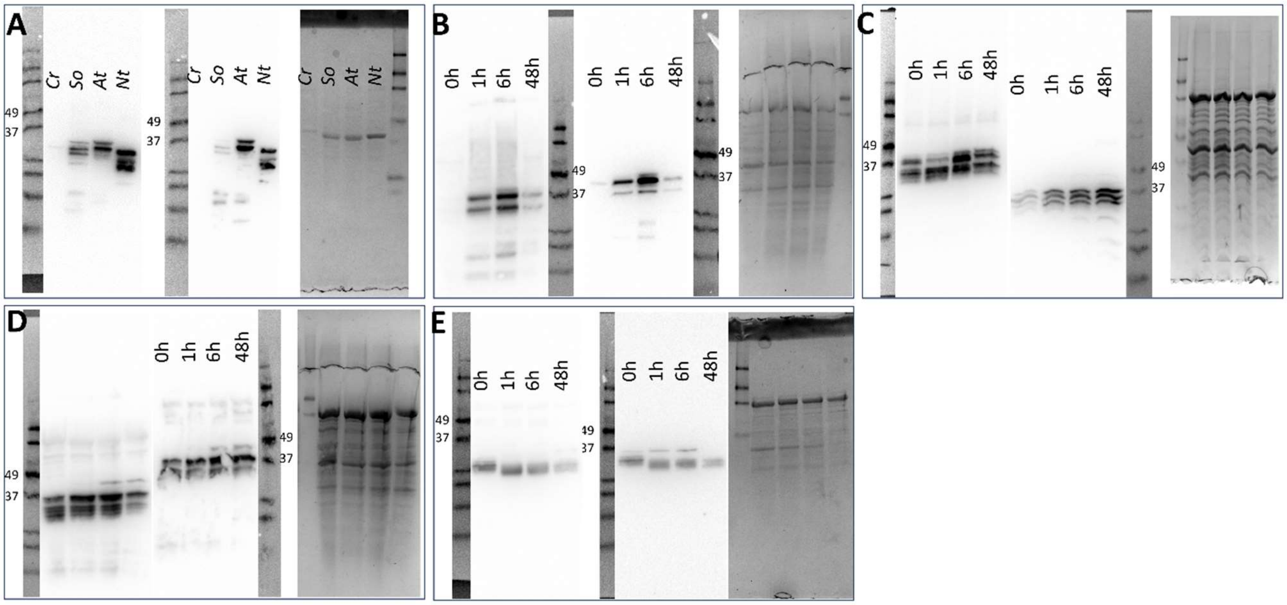

Figure S7: **complete Western blots. A**. controls. **B.** Maize B73. **C.** Maize MR19. **D.** Sorghum. **E.** Setaria. Each panel, left to right: Agrisera antibody, Huabio antibody, Coomassie loading control (panel A, purified protein; panels B-E, total protein before purification). Positions of the 49kDa and 37kDa markers are indicated.

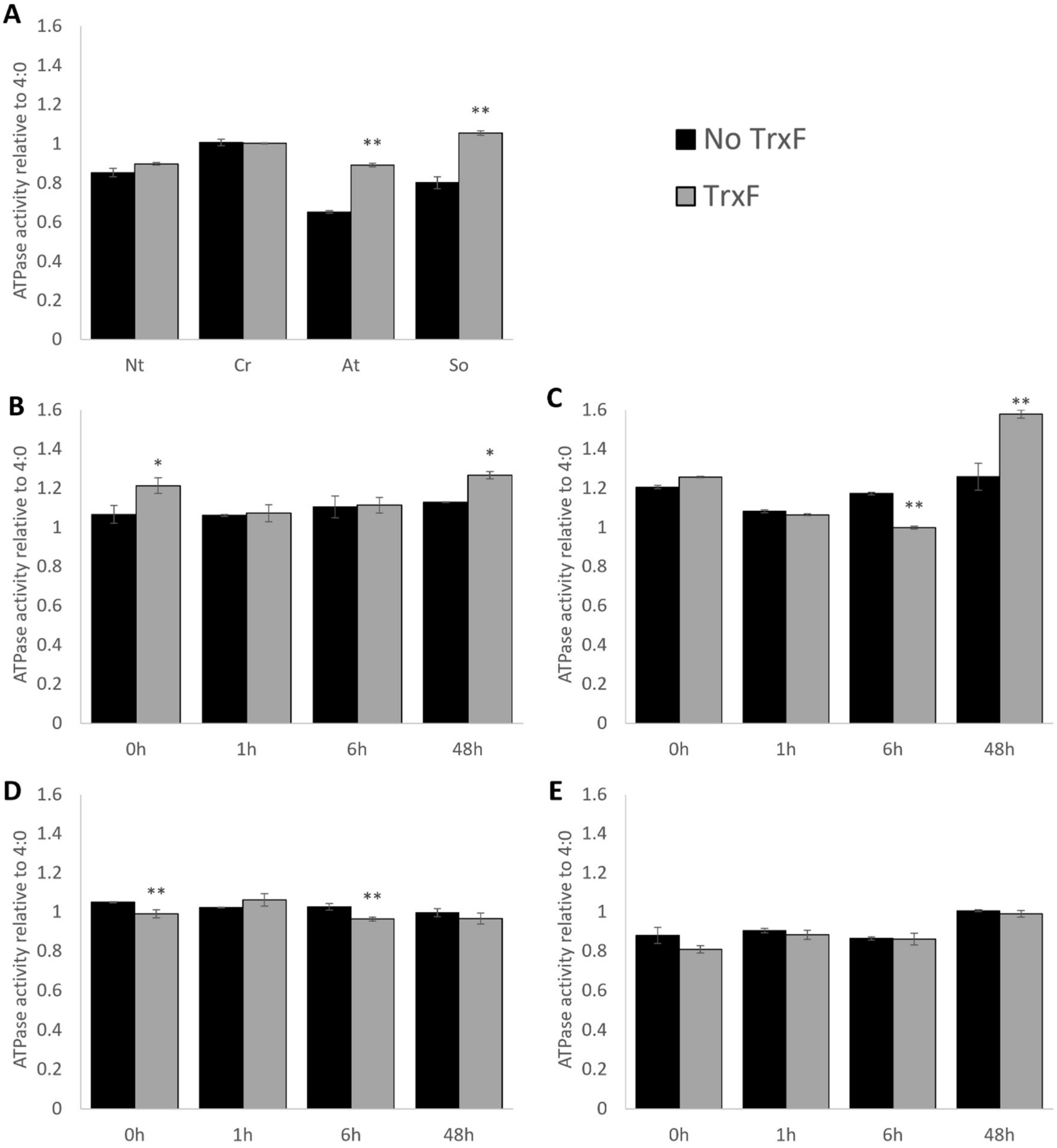

Fig S8: response of ATPase activity to TrxF relative to 4:0 ratio. ATP:ADP ratio was 3:1 and 10mM Mg^{2+}. **A**. Controls **B**. maize B73 **C**. maize MR19 **D**. sorghum **E**. setaria. * $p<0.05$, ** $p<0.01$ vs. no TrxF by Student's T-test, error bars are standard deviation of n=5

We investigated the influence of redox control of RCA activity in the different C_4 plants. RCA contains a disulfide bond present in the CTD of the α isoform, which is reduced by thioredoxin F (TrxF). We therefore tested RCA ATPase activity in the presence and absence of reduced TrxF. We used RCA proteins from Arabidopsis, spinach, tobacco and Chlamydomonas to reproduce published results: stimulation of ATPase activity in Arabidopsis and spinach by TrxF, and the lack of stimulation in both tobacco and Chlamydomonas, both of which lack the α isoform. TrxF-dependent stimulation of ATPase activity was weakly observed in the maize cultivars but does not appear to be relevant in sorghum or setaria.

Genotype	Duration	Ci at 400ppm	Vcmax µmol/m²s	Jmax µmol/m²s	Vpmax µmol/m²s	Rd µmol/m²s	gm µmol/m²s Pa	Stomatal limitation	Limit at 400ppm
B73	0h	129.22	99.13	329.71	205.63	10.00	5.44	0.13	RcPc
B73	0h	118.64	71.64	422.05	111.79	10.00	14.23	0.22	RcPc
B73	0h	113.15	71.67	668.48	163.85	10.00	14.17	0.23	RcPc
B73	48h	212.44	68.72	961.82	279.53	4.81	500.00	0.09	RcPc
B73	48h	99.05	58.78	65`7.85	272.64	6.02	500.00	0.08	RcPc
B73	48h	92.37	58.39	554.02	245.91	1.07	277.80	0.19	RcPc
MR19	0h	100.74	50.28	335.08	157.24	9.89	5.87	0.13	RcPc
MR19	0h	75.92	41.49	507.48	122.16	10.00	4.97	0.01	RcPc
MR19	0h	84.43	101.90	327.62	122.48	9.77	5.24	0.30	RcPc
MR19	0h	128.17	67.23	394.70	110.29	8.97	13.11	0.22	RcPc
MR19	0h	112.56	85.96	285.85	91.05	9.89	23.33	0.25	RcPc
MR19	48h	107.68	63.73	158.43	308.20	10.00	2.02	0.19	RcPc
MR19	48h	131.31	98.27	172.81	320.13	1.00	2.51	0.15	RcPc
MR19	48h	136.91	100.49	186.94	275.65	1.00	4.22	0.15	RcPc
BTx623	0h	72.55	86.31	289.88	127.90	10.00	16.37	0.22	RcPc
BTx623	0h	136.25	56.49	343.50	133.75	10.00	18.04	0.10	**RrPc**
BTx623	0h	62.64	137.50	301.17	312.39	10.00	9.18	0.12	RcPc
BTx623	48h	200.93	236.49	233.70	278.94	10.00	78.47	-0.01	**RrPc**
BTx623	48h	220.73	102.46	252.30	354.08	10.00	118.76	-0.10	**RrPc**
BTx623	48h	153.78	58.76	824.17	344.49	10.00	182.44	0.06	**RrPc**
a10	0h	74.43	62.29	151.04	72.80	1.00	39.51	0.15	**RcPr**
a10	0h	86.27	59.67	177.35	57.49	10.00	13.43	0.22	RcPc
a10	0h	76.02	101.24	163.56	85.83	10.00	2.92	0.26	RcPc
a10	48h	83.19	94.07	242.61	173.54	10.00	138.63	0.27	RcPc
a10	48h	96.40	62.24	413.56	207.83	9.88	166.45	0.24	RcPc
a10	48h	95.82	50.60	826.21	253.84	10.00	157.12	0.20	RcPc

Table S1: A/Ci curve fit values. RcPc: limited by RuBP carboxylation and PEP carboxylation. RrPc: limited by RuBP regeneration and PEP carboxylation. RcPr: limited by RuBP carboxylation and PEP regeneration. Curves that do not follow the RcPc limitation are noted in **red text**.
